# Italian Validation of the Brief Self‐Reported Version of the Spence Children's Anxiety Scale for Children

**DOI:** 10.1002/jclp.70125

**Published:** 2026-03-07

**Authors:** Elide Francesca De Caro, Carlo Garofalo, Elisa Delvecchio, Adriana Lis, Giorgio Ghizzoni, Claudia Mazzeschi

**Affiliations:** ^1^ Department of Philosophy, Social Sciences and Education University of Perugia Perugia Italy; ^2^ Department of Developmental Psychology and Socialization University of Padova Padova Italy

**Keywords:** anxiety, assessment, children, multiple informants, SCAS‐C‐8

## Abstract

**Objectives:**

Clinical assessment of anxiety symptoms in children and adolescents is gaining interest due to the need for brief, valid and reliable instruments that allow early screening through a multiple informant approach. The aim of the present study was to validate the brief self‐reported version of the Spence Children's Anxiety Scale for children (SCAS‐C‐8) by examining its concordances and discrepancies with reports from other informant, for example, parents, as relevant and complementary information for screening.

**Methods:**

Italian children and early adolescents (*N* = 1019; 50.5% female) aged 8–12 years, their mothers and fathers were included in the study by completing the SCAS‐C‐8 and its parent‐version (SCAS‐P‐8). The children and early adolescents also self‐reported internalizing, that is, anxiety and emotional problems, and externalizing symptoms on the other scales.

**Results:**

After confirming the one‐factor structure of the SCAS‐C‐8 and its psychometric properties, that is, reliability, convergent validity with internalizing symptoms and discriminant validity with externalizing symptoms, and complete invariance across sex and age, both concordance and discrepancies between SCAS‐C‐8 and SCAS‐P‐8, were examined. Results confirmed the low/moderate agreement between reports of anxiety and that mothers overestimate levels compared to fathers. Sex and age differences emerged in patterns of discrepancy between reports as well.

**Conclusion:**

Overall, results confirm that the SCAS‐C‐8 is a valid, reliable, brief, and cross‐domain instrument that, together with the parent‐reported version, could help to expand the early detection of anxiety symptoms by adopting a systematic approach with multiple informants capable of integrating relevant and complementary information for prevention and intervention programs.

## 1 Introduction

Anxiety disorders are very common paediatric mental health problems that have increased during and after the COVID‐19 pandemic (Ludwig‐Walz et al. 2023; World Health Organization 2021). Anxiety disorders encompass a wide range of symptoms that can occur as early as childhood and have a high comorbidity in adolescence and even adulthood, negatively impacting quality of life and psychological outcomes throughout life (Dickson et al. 2024). Nevertheless, anxiety‐related symptoms remain under‐recognised by both family members, that is, parents, and the mental health care system (Kara 2022). Several authors agree on two main problems in recognising anxiety symptoms in childhood and adolescence: 1) the overall difficulty in distinguishing the variety of “normal” fears and concerns as part of development from pathological fears that tend to interfere with overall functioning, and 2) the need to validate and systematically implement a cross‐domain anxiety instrument capable of capturing multiple symptoms typical of existing anxiety disorders, that is, separation anxiety disorders, specific phobia, social anxiety disorder, generalized anxiety disorders, and panic disorder, in a brief measurement to favour rapid and early assessment (Delvecchio et al. 2018; Kara 2022; Reardon et al. 2024; Rodríguez‐Menchòn et al. 2022; Waschbusch et al. 2025). In relation to the first point, “normal” anxiety in children has been defined as a developmental response to a specific growth phase, whereas clinically significant anxiety symptoms refer to moderate or elevated levels of anxiety that partially or completely fulfil the diagnostic criteria, that is, they are comparable to normative data, persistent and significantly affect quality of life at different levels (Rapee et al. 2023). For this reason, self‐report of anxiety by children (Van Roy et al. 2010) and accurate assessment with brief and reliable instruments to detect anxiety symptoms is recommended, as these are precursors of later anxiety disorders in the community settings, for example, in primary care and schools for screening purposes (Rapee et al. 2023; Reardon et al. 2019). Most studies of anxiety in children and adolescents use clinical samples, which may limit understanding of the mental health needs, that is, how to recognize and treat the general population. Considering that community samples lead to better generalization of results to the population of children.

Furthermore, valid, reliable and invariant measures of anxiety symptoms would help to systematically investigate the still unclear sex and age differences in anxiety levels (Rapee et al. 2023). Indeed, some previous studies have shown that girls and younger children tend to report more anxiety symptoms compared to boys and adolescents (Bao and Han 2025; Dickson et al. 2024; Delvecchio et al. 2015; Rapee et al. 2023). However, other studies show no differences between sex and age (Rapee et al. 2023). In addition, authors have frequently reported the need to systematically include multiple informants in the assessment and to consider the discrepancies between informants as complementary information that can be used in both screening and clinical settings (De Los Reyes et al. 2023; Navarro et al. 2020; Waschbusch et al. 2025). In particular, previous studies have repeatedly emphasized the need to examine concordance between parents and their children, often finding a pattern of higher concordance between parents, that is, mothers and fathers, and lower concordance with children for internalizing symptoms (Bowers et al. 2019; Hyland et al. 2022). The concordance patterns between parents and children could depend on several factors, which mainly include the sex and age of the children. Overall, previous studies have theorized and found that parents of older children may be less informed about their children's internal states because children begin to interact more with peers, leading to a decrease in reciprocal ratings, while girls are more likely to communicate with parents, which increases ratings (Nauta et al. 2004; Weems et al. 2010). However, the literature on the effects of age and sex on parent‐child agreement in reporting anxiety‐related symptoms is mixed. Some studies have found that agreement increases with child age and that parents tend to report more symptoms of generalized anxiety in younger children than in older children (Hyland et al. 2022; Sequeira et al. 2020), but other studies have found no significant effect of child age (Orgilés et al. 2023). The results on the effects of sex on match are also mixed (Bowers et al. 2019; Sequeira et al. 2020). In some studies, better agreement was found between parents and daughters than between parents and sons (Waschbusch et al. 2025; Weems et al. 2010), while in other studies the opposite pattern or no difference between the sexes was found (Smith et al. 2022). However, the degree of agreement, that is, the simple correlations between parent and child reports, does not necessarily shed light on the discrepancies between the mean anxiety scores reported by mothers, fathers and their children (De Los Reyes et al. 2015). Indeed, the inconsistent results may be due to mixed methods used to examine discrepancies between parents and children, that is, correlations and differences in mean anxiety scores, which are often considered interchangeably. Therefore, discrepancies between multiple informants, that is, agreement degree and differences in mean levels of anxiety reported by mothers, fathers and their children, should be systematically investigated across sex and age groups to examine patterns of under‐ or over‐reported anxiety symptoms (Howard et al. 2025; Hyland et al. 2022). In order to examine whether parents and children report validly, that is, whether the different reports merely reflect the different perspectives of the reporters, the validation of assessment instruments, that is, self‐reports and parental reports, should examine 1) construct validity with other measures in cross‐comparisons (De Los Reyes et al. 2022; Weems et al. 2010) and also 2) whether different patterns of both concordance among the information sources and anxiety related measures, and their construct validity emerge between boys and girls and children and early adolescents. For example, a previous study on agreement between multiple informants found that for younger children, parents were the best informants when assessing anxiety symptoms, followed by the child themselves. However, the authors also emphasized that in older children, the child is often the best informant, followed by the parent, but they did not take sex differences into account. Regarding differences in patterns of construct validity, to our knowledge, only one previous study by Hyland and colleagues examined both patterns of agreement and construct validity between informants and by age of children, with expected differences by age. They found that with increasing cognitive development, children's ability to self‐report their own anxiety level and parents' ability to recognize symptoms decreased as children became more autonomous, particularly in adolescence. To our knowledge, no recent study has addressed such relevant questions about the interpretation of agreement/discrepancy between multiple informants and its validity also considering both sex and age differences, so the present study aims to help fill this gap by examining some of the key features of multiple informant discrepancy in the context of child and adolescent assessment (Hyland et al. 2022; Miller et al. 2014; Smith 2007).

Very few existing instruments are available in their self‐report and other informant versions, such as the parent‐reported version. For example, the Spence Children Anxiety Scale (SCAS; Spence 1997) is one of the most widely used instruments worldwide to assess anxiety in children and adolescents according to the DSM‐IV diagnostic criteria (American Psychiatric Association 1994). The SCAS has been validated in both the child version (SCAS‐C) and the parent version (SCAS‐P) in several child and adolescent populations from different countries (Li et al. 2016; Nauta et al. 2004; Reardon et al. 2019; Spence 1997, 1998).

The possibility to rely on short measures that can be administered in a limited amount of time is critical for multi‐informant research and especially for children‐reports. Recently, a short version (SCAS‐8), which measures several symptoms typical of existing anxiety disorders with only 8 items, demonstrating its suitability for the assessment of anxiety symptoms (Reardon et al. 2018, 2019). In particular, the SCAS‐8 for parents has recently been validated in both the Spanish (Orgilés et al. 2022, 2023) and Italian (De Caro et al. 2025) child and adolescent populations, showing good psychometric properties and convergent validity with the emotional problems and discriminant validity with externalizing symptoms and relationship problems with peers of the Strength and Difficulties Questionnaire (SDQ) (De Caro et al. 2025; Orgilés et al. 2022). However, the SCAS‐8 self‐report for children has only been validated in Spain by Rodrìguez‐Menchón and colleagues (2022), who also demonstrated good psychometric properties. In order to systematically use such an instrument to assess anxiety symptoms in European countries from the perspective of multiple informants and considering cultural differences, further research on the validity of both the parent‐reported and self‐reported SCAS‐8 for children and adolescents as a single‐factor instrument would be required. To our knowledge, no study has yet been conducted to test the validity of the self‐reported SCAS‐8 for children and adolescents in an Italian sample of children and adolescents that does not allow for the integrated use of multiple informant reports (De Los Reyes and Langer 2018; Hunsley and Mash 2007). In addition, given the unclear sex and age differences in self‐reported and parent‐reported anxiety symptoms (Delvecchio et al. 2015; Rapee et al. 2023; Reardon et al. 2025), further support for the validity and sex and age invariance of such a brief instrument for the early detection of anxiety disorders could help to close this gap.

Therefore, the general aim of the present study was to investigate and substantiate the one‐factor structure of the SCAS‐C‐8, its reliability, the association with the SCAS‐P‐8 mothers and fathers related measures in order to systematically account for the multiple‐informant discrepancies, the construct validity with other general and cross‐domains anxiety‐related symptoms, and measurement invariance across sex and age in Italian children and adolescents.

As for the inter‐rater correlations between self‐reported anxiety scores and those assessed by parents, that is, mothers and fathers, they are intended to provide evidence for the use of short and handy instruments that are reliable in assessing anxiety from the perspective of multiple informants. Consistent with De Los Reyes et al. (2023), we expected low to moderate correlation, suggesting that the information provided by children and parents is complementary rather than redundant (De Los Reyes et al. 2023; De Los Reyes et al. 2015; Kaurin et al. 2016). In addition, construct validity and between‐informant estimates, that is, mean differences in self‐ and parent‐ reported levels of anxiety, by age and sex were also examined. Given the mixed results on the role of age and sex on parent‐child agreement, our hypotheses are exploratory: we expect 1) higher parent‐child agreement in younger children compared to older children (Dickson et al. 2024; Sequeira et al. 2020), that is, in early adolescence, 2) better agreement of girls with their parents (Weems et al. 2010), and 3) that associations between informants and other anxiety‐related measures show similar patterns. In addition, we expected that mothers tend to report higher levels of anxiety in young children, while fathers reporting higher levels of anxiety of their daughters compared to sons and young children compared to older one, that is, early adolescents (Hyland et al. 2022; Miller et al. 2014; Smith 2007)

In terms of construct validity and in line with previous studies, we expect to confirm convergent validity (*r* > 0.50) with other general and cross‐domains anxiety‐related measures, that is, trait anxiety, SDQ emotional and peer relationship problems and separation anxiety, respectively (Delvecchio et al. 2015; Delvecchio et al. 2018; Rodríguez‐Menchón et al. 2022), and divergent validity (*r* < 0.30) with SDQ externalizing symptoms related subscales, that is, conduct problems and hyperactivity.

## 2 Materials and Methods

### 2.1 Participants and Procedures

Participants were recruited from private and public cooperative schools serving primarily middle‐class families (absolute SES, Hollingshead 1975). The dean of the school, teachers and parents provided written informed consent prior to inclusion in the study. Children and early adolescents gave verbal consent. No incentives were offered to participants. The study was conducted in accordance with the guidelines of the Declaration of Helsinki and was approved by the the Ethics Committee of the University of Padova for psychological research, of which the authors are members.

Table 1 describes the characteristics of our sample of Italian children and early adolescents (*N* = 1019; *M*_age_ = 10.34, SD = 1.62; 50.5% female) and their mothers and fathers.

**Table 1:** Sample characteristics.

	Total (*N* = 1019)			
Variables	*M*	SD	*n*	%
Children				
Age	10.34	1.62		
Sex				
Female			515	50.5
Male			504	49.4
Age groups				
Children			558	54.8
Early adolescents			461	45.2
Parents				
Age				
Mother	43.18	4.69		
Father	46.01	5.39		

*Note:* Children = 8–10 years old; early adolescents = 11–13 years old.

### 2.2 Measures

#### 2.2.1 The Short Version of the Spence Children's Anxiety Scale for Children (SCAS‐C‐8)

The short version of the SCAS‐C‐8 (i.e., eight items) measures symptoms of social anxiety, separation anxiety, panic/agoraphobia, and generalised anxiety according to the current DSM‐5 (APA, 2013), rated on a 4‐point Likert scale (0‐never, 1‐rarely, 2‐often and 3‐always). The total score ranges from 0 to 24, with higher scores indicating a higher level of anxiety. The correlation with the total score of the SCAS long version was >0.80 (Ahlen et al. 2018).

#### 2.2.2 The Short Version of the Spence Children's Anxiety Scale for Parents (Scas‐P‐8)

The Italian short version of the SCAS‐P (see Table A1 in APPENDIX) with 8 items (SCAS‐P‐8) used in the present study was recently validated by De Caro et al. (2025) by showing good internal consistency (*α* = 0.81). The SCAS‐P‐8 is a parent‐reported measure of anxiety‐related symptoms according to the current DSM‐5 (APA, 2013). Items were rated on a 4‐point Likert scale (0‐never, 1‐rarely, 2‐often and 3‐ always) with a total score ranging from 0 to 24, with higher scores indicating higher level of anxiety.

#### 2.2.3 The State‐Trait Anxiety Inventory for Children (STAI‐C‐T)

The State‐Trait Anxiety Inventory for Children—Trait Scale (STAIC‐T; Spielberger et al. 1973) is a self‐assessment questionnaire containing 20 items measuring anxiety on a 3‐point Likert scale. The Italian version of the instrument was used (Delvecchio et al. 2018). The STAIC‐T has shown good psychometric properties such as a good Cronbach's alpha (0.83) and a good ordinal coefficient (0.87) in an Italian sample of children and early adolescents (8–13 years).

#### 2.2.4 The Separation Anxiety Assessment Scale–Child Version (SAAS‐C)

The Italian version (Delvecchio et al. 2015) of the Separation Anxiety Assessment Scale‐Child Version (SAAS‐C; Hahn et al. 2003) is a 34‐item self‐report scale that measures four symptom dimensions of separation anxiety disorder (SAD), which are subdivided into four 5‐item subscales, that is, fear of abandonment, fear of being alone, fear of physical illness, and worry about calamitous events. The SAAS‐C showed a good Cronbach's alpha (0.83). For the present study, the total score of the SAAS‐C was calculated and used.

#### 2.2.5 The Strength and Difficulties Questionnaires for Children (SDQ)

The Italian version (Riso et al. 2010) of the SDQ for children (Goodman et al. 1998) was used to assess their difficulties and strengths. This scale contains 25 items divided into five subscales and rated on a three‐point scale (from ‘0 = *strongly disagree*’ to ‘2 = *strongly agree*’). The SDQ contains five subscales: emotional problems, conduct problems, hyperactivity, peer relationship problems, and pro‐social behaviour. Previous studies have found, the Cronbach alpha for the emotional problems subscales ranging from 0.57 to 0.66 (Capron et al. 2007; Di Riso et a., 2010; Muris et al. 2004).

### 2.3 Data Analysis

All data were processed with IBM SPSS.25 (IBM Corp Released 2017) and the statistical framework R (R Core Team 2019) within the software JASP Team (2024).

Confirmatory factor analysis (CFA) was performed to test the factor structure of the items using the Lavaan package (Rosseel 2012) for R. A robust estimator (WLSMV) was used. Several indices were used to test the goodness of fit of the model. Hair et al. (2013) recommended that factor loading indices should be higher than 0.50 (ideally ≥ 0.70) but a value above 0.40 may be acceptable in exploratory studies. The structural equation model was used to assess measurement invariance across sex and age by comparing successive levels of measurement invariance with additional and more progressive equality constraints, respectively: configural, metric, and scalar (Putnick and Bornstein 2016). In line with recent guidelines for testing invariance (Wu and Estabrook 2016; Tse et al. 2024), strict invariance (i.e., equality of loadings, intercepts and residual variances) was also tested. To establish measurement invariance the following criteria (Bikos 2024; Chen 2007; Putnick and Bornstein 2016) were considered: ∆χ^2^, *p* > 0.05, ∆CFI ≤ 0.01, ∆TLI ≤ 0.01, ∆RMSEA ≤ 0.015 and ∆SRMR ≤ 0.03 (for metric invariance) or ∆SRMR ≤ 0.015 (for scalar and strict invariance). Changes in fit indices greater than these values were considered indicative of non‐invariance. Cronbach' alpha coefficient was used to check the internal consistency of the SCAS‐C‐8 factor. The factor score was calculated to 1) compare multiple informants' reports and estimate mean differences in self‐ and parent‐reported anxiety by children's sex and age, and 2) determine construct validity, that is, concurrent and divergent validity, in the context of correlations between the factor and children's reports of the SDQ subscales and other anxiety‐related dimensions, state‐trait anxiety (STAIC‐Trait), and separation anxiety. In addition, construct validity was examined in the context of multiple informants' reports. The *95% confidence interval (CI)* for mean differences was also reported, and the effect size of differences in anxiety ratings across informants was estimated using both *Eta squared (▯* ^*2*^ *)* for the proportion of variance explained by between‐group differences and *Cohen's d (d)* for the magnitude of between‐group mean differences (small = 0.2, medium = 0.5, or large = 0.8; LeCroy and Krysik 2007). Finally, we provided a contribution to the sensitivity of the SCAS‐C‐8 as a brief and reliable instrument capable of discriminating groups by presence/absence of anxiety symptoms measured with the STAI‐C trait (Siddaway et al. 2018) above and below its cutoff that is 40 (Ercan et al. 2015). We present the results in Table S1.

## 3 Results

### 3.1 Missing Data

No missing data were found for the items of the SCAS‐C‐8 and STAI‐C trait. We analysed the patterns of missing values on the SCAS‐8 parent reports, SAAS and SDQ‐related items. The patterns for the items showed that missing responses to individual items ranged from 1.1% (SCAS‐P‐8 maternal reports) to 2.6% (SAAS) of the total responses given: the pattern of cases where there were no missing values was the most widespread (88.8% to 99.9%). The Missing Completely at Random (MCAR) test indicated that our data for the SCAS‐P‐8 parent report, the SAAS, and the SDQ were not completely random (*p* < 0.001), but we verified that no item pairs tended to have missing values in individual cases. Therefore, we used the Expectation Maximization (EM) method to estimate replacement values for each missing item and calculate the scale scores for each participant.

### 3.2 Confirmatory Factor Analysis of SCAS‐C‐8

A confirmatory factor analysis (CFA) of SCAS‐C‐8 related items was run according to the validation by Rodríguez‐Menchón et al. (2022). The results showed that the one‐factor model reached a good fit (*χ* ^*2*^ = 62.89, *df* = 20, *p* < 0.001; CFI = 0.96; TLI = 0.94; RMSEA = 0.05; 95% CI [0.03–0.06]; SRMR = 0.05). Table 2 shows both the unstandardized and standardized factor loadings estimate. All standardized factor loadings were above or equal 0.40. SCAS‐C‐8 showed an acceptable alpha coefficient of 0.70.

**Table 2:** Confirmatory factor analysis.

Item	Estimate	SE	*Z*	Stand. Estimate
1. I worry about things	0.31	0.03	10.86	0.44
2. I feel afraid	0.33	0.03	11.58	0.50
3. I worry about being away from my parents	0.50	0.04	14.39	0.56
4. I feel scared if I have to sleep on my own	0.35	0.03	10.09	0.50
5. I have trouble going to school in the mornings because I feel nervous or afraid	0.30	0.03	8.39	0.40
6. I suddenly start to tremble or shake when there is no reason for this	0.33	0.03	10.44	0.44
7. I worry that I will suddenly get a scared feeling when there is nothing to be afraid of	0.36	0.03	11.55	0.74
8. I would feel scared if I had to stay away from home overnight	0.34	0.04	9.56	0.40

*Note:* Factor loadings estimate of items of SCAS‐C‐8. SCAS‐C‐8 = Spence Children's Anxiety Scale for Children; Estimate = unstandardized factor loadings; Stand. Estimate = standardized factor loadings; All estimates are at *p* < 0.001.

### 3.3 Measurement Invariance of SCAS‐C‐8 Across Sex and Age Groups

The invariance of the one‐factor model of SCAS‐C‐8 was tested across sex and age. The results showed that full configural, metric, scalar and strict invariance was confirmed for both sex and age (Table 3). Given the good measurement invariance across sex and age, we performed comparisons of the means of the latent factor across sex and age. A one‐way ANOVA was then performed to examine the mean differences between the sexes and ages of the children in both self‐reported and parent‐reported anxiety scores. The results show both sex and age‐related differences in self‐reported mean anxiety scores, that is, girls were found to systematically report higher mean scores on the SCAS‐C‐8 factor and young children higher mean scores compared to early adolescents in both sex groups (Table 4).

**Table 3:** Measurement invariance across sex and age: Multi‐group CFA.

	Model	χ^2^(*df*)	CFI	TL1	RMSEA [90% CI]	SRMR	Δχ^2^_(DF)_	ΔCFI	ΔRMSEA	ΔSRMR
**Sex**	Config. Invariance	77.152(38)	0.96	0.94	0.05 [0.03–0.06]	0.05				
	Metric Invariance	83.978(45)	0.96	0.95	0.04 [0.03–0.05]	0.05	6.826(7)	0.00	0.01	0.01
	Scalar Invariance	83.978(53)	0.97	0.96	0.03 [0.02–0.05]	0.05	0.000(8)	0.01	0.01	0.01
	Strict Invariance	84.217(61)	0.97	0.98	0.03 [0.01–0.04]	0.05	0.239(8)	0.00	0.00	0.00
**Age**										
	Config. Invariance	71.381(38)	0.97	0.95	0.04 [0.03–0.06]	0.05				
	Metric Invariance	85.249(45)	0.96	0.95	0.04 [0.03–0.06]	0.05	13.868(7)	0.01	0.00	0.00
	Scalar Invariance	85.249(53)	0.97	0.96	0.04 [.02‐.05]	0.05	0.000(8)	0.01	0.00	0.00
	Strict Invariance	86.340(61)	0.97	0.98	0.03 [0.01–0.04]	0.05	1.091(8)	0.00	0.01	0.01

*Note: N* = 1019; Male = 504; Female = 515; children (8–10 yrs.) = 558; Early adolescents (11–13 yrs.) = 461; χ^2^ = Chi‐square statistic; CFI = Comparative fit indices (>95); TLI = Tucker–Lewis Index (>95); RMSEA = Root Mean Square Error of Approximation (<08); SRMR = Standardized Root Mean Square Residual (<0.08).

**Table 4:** Mean, standard deviations of SCAS‐C‐8, SCAS‐P‐8 Mother and Father: Age and sex comparisons.

	Total sample *N* = 1019				8–10 yrs. old (*n* = 558)	11–13 yrs. old (*n* = 461)	One‐way ANOVA F_(_ղ^2^_)_	
	*M* (SD)	*α*	M (*n* = 504)	F (*n* = 515)	M (SD)	M (SD)	F_SEX_ (ղ^2^)	F_AGE_ (ղ^2^)
SCAS‐C‐8	5.47 (3.38)	0.70	4.94 (3.31)	5.98 (3.38)	6.07 (3.67)	4.75 (2.84)	39.44*** (0.04)	23.07*** (0.02)
SCAS‐P‐8 Mother	8.17 (2.70)	0.60	8.00 (2.56)	8.32 (2.83)	8.38 (2.77)	7.89 (2.59)	3.51 (0.00)	8.02** (0.01)
SCAS‐P‐8 Father	7.51 (2.55)	0.58	7.25 (2.58)	7.75 (2.50)	7.67 (2.62)	7.03 (2.47)	9.83** (0.01)	4.81* (0.01)
RM ANOVA F_(_ղ^2^_)_	363.15 (0.26)***		6.46 (0.00)**		12.57 (0.01)***			

*Note:* SCAS‐C‐8 = Spence Children's Anxiety Scale for Children; SCAS‐P‐8 = Spence Children's Anxiety Scale for Parents; RM = Repeated Measure

* *p* < 0.05; F = Fisher‐Snedecor test; ղ^2^ = eta square

** *p* < 0.01;

*** *p* < 0.001.

### 3.4 Comparisons Between Multiple Informants: Reliability Coefficients, Mean Differences in Self‐ and Parent‐Reported Anxiety by Sex and Age of Children

Table 4 contains mean values and standard deviations for SCAS‐C‐8, SCAS‐P‐8 mothers and fathers. A one‐way ANOVA was then performed to examine the mean differences between the sexes and ages of the children in parent‐reported anxiety scores. The results show sex differences in the reports of fathers reporting higher mean scores for female children (Table 4).

A repeated measures ANOVA was then used to compare reports across informants. Significant mean differences between SCAS‐C‐8 and SCAS‐P‐8 mothers (mean differences = −2.69, *SE* = 0.12, *t* = −23.39, *df* = 1018, *d* = −0.93, *95% CI* [−1.03, −0.82], *p* < 0.001) and fathers (mean differences = −2.04, *SE* = 0.12, *t* = −17.69, *df* = 1018, *d* = ‐0.70, *95% CI* [−0.80, −0.59], *p* < 0.001), indicating that mothers tended to overreport their children's level of anxiety compared to self‐reported and father‐reported anxiety (mean differences = 0.66, *SE* = 0.08, *t* = 8.41, *df* = 1018, *d* = 0.23, *95% CI* [0.16, 0.29], *p* < 0.001).

**Figure 1:**
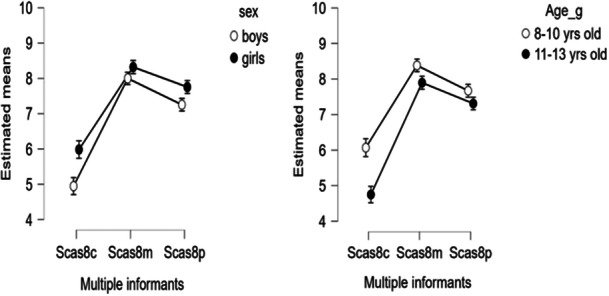
Estimated mean differences between self‐ and parents‐reported anxiety by sex and age of children. Estimated means: Repeated measures (RM) ANOVA estimated means by sex and age; Scas8c = SCAS‐C‐8 children reports; Scas8m = SCAS‐P‐8 mothers reports; Scas8p = SCAS‐P‐8 fathers reports; The figures display the interaction effect Multiple informants SCAS‐8 by sex and age (Table 4).

The results (Table 4) also show the gender and age differences of the children in the mean differences between the parents and the self‐assessments. Both mothers (Mean differences by age = −2.73, *SE* = 0.12, *t* = 23.76, *df* = 1017, *d* = ‐0.95, *95% CI* [−1.04, −0.83], *p* < 0.001; Mean differences by sex = −2.69, *SE* = 0.12, *t* = −23.52, *df* = 1017, *d* = ‐0.93, *95% CI* [−1.06, 0.84], *p* < 0.001) and fathers (Mean differences by age = −2.08, *SE* = 0.12, *t* = −18.15, *df* = 1017, *d* = ‐0.72, 95% CI [−0.83, −0.62], *p* < 0.001; mean differences by sex = −2.03, *SE* = 0.12, *t* = 17.75, *df* = 1017, *d* = −0.70, 95% CI [−0.81, −0.60], *p* < 0.001) tend to overestimate anxiety symptoms in younger children compared to early adolescents and in their daughters compared to sons (Figure 1).

### 3.5 Concordance Between Self‐ and Parent‐ Reported Anxiety and Construct Validity Related Patterns Across Informants and by Children's Sex and Age

Simple correlations between SCAS‐C‐8 and SCAS‐P‐8, mothers and fathers and the STAIC state and trait scales, the SDQ‐EMO and SAAS scales for convergent validity, and other SDQ subscales, that is, conduct, hyperactivity, and relationship problems with peers, for discriminant validity were examined in the total sample and by sex and age.

Table 5 also shows high significant correlations between the SCAS‐C‐8 and the STAI‐C trait anxiety scales as well as the SAAS scales, while low to moderate correlations were found with the SDQ Emotional Problems. In addition, the results show stronger construct validity of the SCAS‐C‐8 compared to parent reports, which show poor or no correlations with all other related measures (Table 5).

**Table 5:** Simple correlations among SCAS‐C‐8, SCAS‐P‐8 mothers and fathers, and SDQ subscales, STAI‐T and SAAS, for total sample, sex and age groups.

	Total sample							
	SCAS‐P‐8 Mother	SCAS‐P‐8 Father	SDQ EMO	SDQ CON	SDQ HYP	SDQ PEER	STAI‐T	SAAS
1 SCAS‐C‐8 Pearson's *r* 95% CI								
	0.29** [0.22–0.35]	0.26** [0.20–0.32]	0.52** [0.47–0.57]	0.23** [0.17–0.29]	0.20** [0.13–0.26]	0.20** [0.13–0.26]	0.63** [0.59‐.66]	0.25** [0.18–0.32]
2. SCAS‐P‐8 Mother Pearson's *r* 95% CI								
		0.56** [0.51–0.60]	0.28** [0.22–0.34]	0.09** [0.02–0.15]	0.05 [−0.01–0.12]	0.14** [0.08–0.20]	0.25** [0.18‐0.31]	0.28** [0.22–0.34]
3. SCAS‐P‐8 Father Pearson's r 95% CI								
			0.25** [0.20–0.31]	0.00 [0.00–0.03]	0.00 [−0.06–−0.06]	0.11** [0.05–0.17]	0.21** [0.15–0.26]	0.24** [0.18–0.30]
Sex								
1								
Girls	0.28**	.23**	0.50**	0.25**	0.18**	0.22**	0.60**	0.64**
Boys	(0.29**)	(0.27**)	(0.51**)	(0.25**)	(0.24**)	(0.20**)	(0.64**)	(0.66**)
2								
Girls		0.54**	0.25**	0.11*	0.06	0.11*	0.26**	0.28**
Boys		(0.57**)	(0.30**)	(0.07)	(0.05)	(0.18**)	(0.22**)	(0.27**)
3								
Girls			0.22**	0.04	0.03	0.09*	0.19**	0.22**
Boys			(0.27**)	(−0.01)	(−0.01)	(0.14**)	(0.20**)	(0.25**)
Age groups								
1								
Children	0.30**	0.26**	0.47**	0.17**	0.18**	0.15**	0.61**	0.67**
Early adolescents	(0.24**)	(0.25**)	(0.57**)	(0.27**)	(0.20**)	(0.21**)	(0.66**)	(0.59**)
2								
Children		0.57**	0.27**	0.12**	0.05	0.14**	0.23**	0.29**
Early adolescents		(0.53**)	(0.28**)	(0.02)	(0.05)	(0.10*)	(0.26**)	(0.23**)
3								
Children			0.24**	0.02	−0.01	0.09*	0.19**	0.23**
Early adolescents			(0.25**)	(0.03)	(0.00)	(0.10* ^)^	(0.23**)	(0.22**)

*Note: N* = 1019; Male = 504; Female = 515; children (8–10 yrs.) = 558; Early adolescents (11–13 yrs.) = 461; SCAS‐C‐8 = Spence Children's Anxiety Scale for Children; SCAS‐P‐8 = Spence Children's Anxiety Scale for Parents; SAAS‐C = The Separation Anxiety Assessment Scale–Child Version; STAIC‐T = State‐Trait Anxiety Inventory for Children Trait subscale; the correlations refer to girls and young children while the correlations in round brackets refer to boys and adolescents.

* *p* < 0.05

** *p* < 0.01.

Regarding patterns of construct validity by sex and age of children, higher convergent validity of SCAS‐C‐8 was found for early adolescents with the SDQ Emotional Problems compared to younger children (*z* = −2.18, *p* < 0.05), while higher convergent validity was found with SAAS for younger children compared to early adolescents (*z* = 2.11, *p* < 0.05), while no sex and age differences were found for discriminant validity, with overall low correlations found between the SCAS‐C‐8, the SDQ subscales of conduct, hyperactivity and relationship problems with peers. No sex and age differences were found in the patterns of construct validity of cross‐informants.

### 3.6 Sensitivity of the SCAS‐C‐8 in Distinguishing Groups With/Without Anxiety

A one‐way ANOVA was conducted to examine the mean differences in SCAS‐C‐8 factor scores between children and early adolescents with anxiety scores above and below the STAI‐C trait cut‐off of 0.40 (Ercan et al. 2015). Results showed significant SCAS‐C‐8 mean differences with larger effect sizes for self and mother reports (see supporting material for details).

## 4 Discussion

The early identification of anxiety disorders in children and adolescents is an important topic for empirical research, which needs to include valid, brief, and cross‐domain assessment instruments for a wide range of anxiety symptoms such as separation anxiety, specific phobia, social anxiety disorder, generalised anxiety disorder, and panic disorder. The aim of the present study was to contribute to the validation of the brief and self‐reported version of the SCAS‐C‐8 for assessing cross‐domain anxiety symptoms in children and adolescents by including discrepancies with the reports of other informants, such as parents, as relevant and complementary information for screening (De Los Reyes et al. 2022; De Los Reyes and Kazdin 2004; Weems et al. 2010).

First, in line with Rodrìguez‐Menchón and colleagues (2022), our results helped validate the one‐factor structure of the SCAS‐C‐8 by demonstrating its reliability and its full configural, metric, scalar, and strict measurement invariance across sexes and age, indicating the suitability of the instrument for different demographic groups and allowing systematic comparisons of observed scores on the latent construct. Comparison of mean scores consistently revealed significant sex and age differences in self‐reported anxiety scores, with girls and younger children, that is, aged 8 to 12 years, reporting higher levels of anxiety compared to boys and early adolescents (Bao and Han 2025; Delvecchio et al. 2015; Dickson et al. 2024; Rapee et al. 2023). In line with previous studies, our results also supported construct validity by showing convergent validity with high positive correlations with anxiety traits and emotional problems, but low convergent validity with more specific symptoms such as separation anxiety and discriminant validity with behaviour, hyperactivity, and relationship problems with peers (De Caro et al. 2025; Orgilés et al. 2022). When examining differences in the construct validity of self‐reports, we found stronger convergent validity for emotional problems in early adolescents compared to younger children, which is consistent with cognitive development, that is, a higher ability to self‐evaluate internal states and to discriminate anxiety symptoms, while higher convergence validity for separation anxiety, that is, SAAS, was found in younger children, indicating a distinct and recognizable state of separation anxiety in children (Hyland et al. 2022).

Second, agreement between multiple informants, that is, children and their parents, both mothers and fathers, was examined by testing correlations and mean differences between reports (De Los Reyes et al. 2022; De Los Reyes and Kazdin 2004). In line with previous studies (De Los Reyes et al. 2023; De Los Reyes et al. 2015; Kaurin et al. 2016), the results supported the low to moderate agreement between self‐reports and parental reports of anxiety symptoms, that is, mothers' and fathers' reports, by further confirming that the information provided by these multiple informants is complementary rather than redundant with no different patterns in relation to children's sex and age differences. Despite the higher agreement between mothers' and fathers' reports, when mean differences between reports were examined, results showed that mothers tended to overestimate anxiety scores compared to fathers when compared to their children's self‐reports, suggesting a likely effect of observational context that may differ between informants, that is, mothers spending more time with children than fathers (De Los Reyes et al. 2015, 2022). The results of the study may help to underpin the relevance of the multiple informant approach to assessing anxiety symptoms, in which self‐reports should be systematically considered alongside other people's reports, as they complement rather than overlap. This was also supported by the cross‐comparison of construct validity, which revealed stronger construct validity of self‐reports compared to parent reports in the SCAS‐8, supporting the consistency of children's ability to report their own emotional states. Such cross‐informant patterns of construct validity were stable to reveal informants' own perspectives across children's sex and age, as no differences were found (De Los Reyes et al. 2022; Hyland et al. 2022; Weems et al. 2010). In contrast to the results of the earlier study by Hyland et al. (2022), which found age differences in the construct validity patterns of parental reports indicating greater parental difficulty in recognizing anxiety symptoms than the greater autonomy of adolescence, we found no differences. This could be since children are not yet as independent, but rather dependent on their parents (Hyland et al. 2022).

Third, patterns of agreement between informants and estimates of mean differences by sex and age of children were further explored. The results helped to confirm sex‐ and age‐related differences in mean scores by showing that mothers tended to report higher levels of anxiety in younger children and fathers in both their daughters and younger children than was the case in early adolescents and their sons, respectively, and shed light on differences in relationships between fathers and daughters and fathers and sons in terms of emotional problems (Bowers et al. 2019; Sequeira et al. 2019; Smith et al. 2022; Waschbusch et al. 2025).

When examining mean differences between informant reports, that is, children, mothers and fathers, on levels of anxiety by sex and age of children, results showed that both mothers and fathers tended to overestimate levels of anxiety compared to self‐reports for female and younger children, suggesting that some sex‐ and age‐specific aspects may occur in the relationship between fathers and daughters and need to be considered in future research. This is partly at odds with previous studies which have found that for younger children, the best informants are the parents and the children themselves (Miller et al. 2014; Smith 2007). Overall, our findings help to fill a gap in relation to sex and age differences in parental reporting of anxiety symptoms, which should be systematically considered both in the validation of anxiety assessment instruments and in the clinical context when interpreting results (De Los Reyes et al. 2022; Smith 2007), although they remain to be replicated.

Finally, we found high mean differences of the SCAS‐C‐8 between the groups with and without anxiety symptoms according to the STAI‐C trait scales, which specifically assess the presence or absence of anxiety in children and contribute to the performance and clinical sensitivity of the instrument.

### 4.1 Limitations

Despite the contribution of our study to the validation of the SCAS‐8‐C, a brief instrument for the early detection of anxiety symptoms in children, there are some limitations. First, we accounted for measurement invariance by age as a categorical variable, as this is discontinue since the children are between 8 and 13 years old and thus belong to two different developmental stages, namely middle childhood (8‐10 years) and early adolescence (11–13 years), and two different school levels, namely primary and lower secondary school, in line with previous studies (Cliffordson 2010). However, it is worth noting that a continuum approach would be useful to examine the effects of age in longitudinal studies and to test an assumed continuity of anxiety across age. Second, we did not include other family‐related factors such as parenting and information such as mothers' and fathers' daily routines as relevant variables affecting the sex and age differences in cross‐informant perspectives and concordance patterns findings (De Los Reyes et al. 2023).

Finally, it may be useful for the future to introduce a longitudinal design to further validate the multiple informants approach based on the SCAS‐8 reports to have the possibility to examine nested data developmentally, that is, across different ages, and across situations, that is, considering dyads or nested family data (Hyland et al. 2022).

## 5 Conclusion

These limitations notwithstanding, the present study goes beyond replication of previous findings by contributing incremental knowledge on several grounds. First, is the first to examine the validity of the SCAS employing a multi‐informant perspective, hence supporting the validity of self‐reports of anxiety symptoms in children and adolescents, while also providing insight into parents' contribution to the assessment of their children's anxiety symptoms. These findings emphasize the need to consider both qualitative and quantitative aspects of individual functioning, such as parents' psychological symptoms and well‐being, their relationships, and the context of observation, that is, time spent with the child, time spent with the child, acceptance, communication, and openness (Weems et al. 2010), which could support the interpretation of sex‐ and age‐specific patterns of matches and mismatches and the screening decision‐making process (De Los Reyes et al. 2022; De Los Reyes 2011; Treutler and Epkins 2003; Weems et al. 2010).

Second, our study contributes to provide support for SCAS‐C‐8 as a valid, reliable, brief and cross‐informants tools, expanding its construct validity and known nomological network to a wider range of external correlates including different measures of anxiety as well as internalizing/externalizing symptoms and protective factors. These findings might aid future research into the standardization process of correspondence and discrepancies in mean scores levels of anxiety symptoms. This is in line with suggestions by De Los Reyes et al. (2022) to define a practice for investigating and interpreting the role of such different perspective on symptoms in predicting psychological symptoms, that is, internalizing and externalizing symptoms, and including cross‐contextual determination of reports by considering specific qualitative and quantitative aspects related to the home‐specific (parent) and other such as school‐specific (teacher) informants (De Los Reyes et al. 2022; Shemmassian and Lee 2016).

Finally, we provided a contribution to the sensitivity of the SCAS‐C‐8 as a self‐report anxiety measure capable of discriminating between groups with and without anxiety symptoms according to the STAI‐C trait scales. The results are relevant for screening in both psychological and medical contexts, that is, for the assessment of anxiety before and after a medical intervention and for the evaluation of the effectiveness of psychological interventions to reduce anxiety as well as for intervention practice (Ates Besirik and Canbulat Sahiner 2024; Sahiner et al. 2025; Tuncay and Sarman 2024; Türkmen et al. 2022). Future studies should include a clinical group for comparisons to further test the clinical sensitivity of SCAS‐C‐8 and perform a more sophisticated analysis.

In conclusion, our study makes an additional contribution to the validation of a brief instrument that can assess various anxiety symptoms in children and adolescents with a single measurement tool, as a valid and more efficient assessment of anxiety symptoms is needed in research and in application. Our investigation takes into account parental reports of anxiety symptoms by including both mothers and fathers, as the latter have not often been included in studies with multiple informants, and provides new insights into differences in agreement patterns and construct validity that may be relevant for future screening and intervention practice.

## Funding

The authors received no specific funding for this work.

## Ethics Statement

The study was conducted according to the guidelines of the Declaration of Helsinki and approved by the Ethics Committee of the University of Padova.

## Conflicts of Interest

The authors declare no conflicts of interest.

## Supporting information


**Table S1:** Estimated mean differences of the SCAS‐8 self‐reports and parental reports between the groups with and without anxiety symptoms according to STAI‐C traits.

## Data Availability

The data that support the findings of this study are available from the corresponding author upon reasonable request.

## 1

**Table A1:** By De Caro et al. (2025) factor loadings estimate of items of SCAS‐P‐8.

Item	Estimate	SE	*Z*	Stand. estimate
1. Worries about things	0.41	0.03	15.67	0.62
2. Feeling afraid	0.47	0.02	19.64	0.73
3. Worries about being away from us	0.41	0.03	13.08	0.54
4. Afraid will make fool of self	0.39	0.04	10.95	0.46
5. Trouble going to school in mornings	0.39	0.04	10.26	0.47
6. Worries something bad happen to her/him	0.51	0.03	16.49	0.72
7. Worries that others think of him/her	0.42	0.04	11.91	0.49
8. All of a sudden feel really scared for no reason	0.41	0.03	12.41	0.57

*Note:* Estimate = unstandardized factor loadings; Stand. Estimate = standardized factor loadings; All estimates are at *p* < 0.001.
