# Supplementary material for: Italian Validation of the Brief Self‐Reported Version of the Spence Children's Anxiety Scale for Children
Source: J Clin Psychol. 2026 Mar 7;82(6):948–59. doi: 10.1002/jclp.70125 (PMC13127843; doi:10.1002/jclp.70125)
Supplement: Supplementary file 1 — Table S1: Estimated mean differences of the SCAS‐8 self‐reports and parental reports between the groups with and without anxiety symptoms according to STAI‐C traits. [file JCLP-82-948-s001.docx]

Supplementary material

**Table S1:** *Estimated mean differences of the SCAS-8 self-reports and parental reports between the groups with and without anxiety symptoms according to STAI-C traits*

|  | STAI-C-T  Presence  (*N*=190) | STAI-C-T  Absence  (*N*=829) |  |  |  |  |  |  |  |  |
| --- | --- | --- | --- | --- | --- | --- | --- | --- | --- | --- |
| SCAS-8 | *M (SD)* | *M (SD)* | *t* | *F* | *Mean differences*  *(SE differences)* | *95% CI*  *for mean differences* | *d* | *SE* | *ⴄ^2^* | *p* |
| Self-reports | 8.76 (3.55) | 4.72 (2.85) | -16.76 | 280.96 | -4.04 (0.24) | [-4.51, -3.57] | -1.35 | 0.09 | 0.23 | < .001 |
| Mother-reports | 9.23 (2.93) | 7.92 (2.59) | -6.15 | 37.84 | -1.32 (0.21) | [-1.73, -0.89] | -0.49 | 0.08 | 0.04 | < .001 |
| Father-reports | 8.19 (2.55) | 7.35 (2.53) | -4.11 | 16.89 | -0.83 (0.20) | [-1.24, -0.44] | -0.33 | 0.08 | 0.02 | < .001 |

*Note.* STAIC-T= State-Trait Anxiety Inventory for Children Trait subscale; Presence = anxiety presence; Absence = anxiety absence; SCAS-8= Spence Children’s Anxiety Scale 8 items version; M = mean; SD = standard deviation; SE = standard error; t = Student’s t test; d = Cohen’s d; F = Fisher-Snedecor test; ղ^2^ = eta square; DF = degrees of freedom are 1017 for the variables; CI = Confidence Interval; *** p < .001, ** p < .01, * p <.05
